# Oxidative Stress and Bio-Regulation

**DOI:** 10.3390/ijms25063360

**Published:** 2024-03-15

**Authors:** Toshikazu Yoshikawa, Fukka You

**Affiliations:** 1 Louis Pasteur Center for Medical Research, Tanakamonzen-cho 103-5, Sakyo-ku, Kyoto 606-8225, Japan; 2 Department of Gastroenterology and Hepatology, Graduate School of Medical Science, Kyoto Prefectural University of Medicine, Kajii-cho, Kawaramachi-Hirokoji, Kamigyo-ku, Kyoto 602-8566, Japan; 3 Division of Anti-Oxidant Research, Life Science Research Center, Gifu University, Yanagito 1-1, Gifu 501-1194, Japan; y@antioxidantres.jp; 4 Anti-oxidant Research Laboratory, Louis Pasteur Center for Medical Research, Tanakamonzen-cho 103-5, Sakyo-ku, Kyoto 606-8225, Japan

**Keywords:** oxidative stress, anti-oxidant, free radicals, biochemistry, bio-regulation, reactive oxygen species

## Abstract

Reactive oxygen species (ROS) and free radicals work to maintain homeostasis in the body, but their excessive production causes damage to the organism. The human body is composed of a variety of cells totaling over 60 trillion cells. Each cell performs different functions and has a unique lifespan. The lifespan of cells is preprogrammed in their genes, and the death of cells that have reached the end of their lifespan is called apoptosis. This is contrary to necrosis, which is the premature death of cells brought about by physical or scientific forces. Each species has its own unique lifespan, which in humans is estimated to be up to 120 years. Elucidating the mechanism of the death of a single cell will lead to a better understanding of human death, and, conversely, the death of a single cell will lead to exploring the mechanisms of life. In this sense, research on active oxygen and free radicals, which are implicated in biological disorders and homeostasis, requires an understanding of both the physicochemical as well as the biochemical aspects. Based on the discussion above, it is clear to see that active oxygen and free radicals have dual functions of both injuring and facilitating homeostasis in living organisms.

## 1. Introduction

Oxygen, an essential gas for living organisms, has been known to be toxic since its discovery. However, in the 1950s, Rebecca Gerschman [1] and her colleagues noticed that oxygen poisoning resembled damage caused by X-rays and proposed the involvement of free radicals, which greatly advanced our understanding of the mechanism [2].

Later, based on radiation chemistry, researchers proposed the possibility that free radicals are also involved in aging [3]. However, the modern history of redox biology began with the discovery of superoxide dismutase (SOD) by Joe M. McCord and Irwin Fridovich [4]. Although the initial focus was on free radicals and macromolecular damage, over time, electrophilic reagents derived from hydrogen peroxide (H_2_O_2_) and lipid peroxidation or metabolism, produced by hydroperoxides, especially NADPH oxidase, were recognized to play an important role in physiologically relevant signal transduction [5,6]. The signaling mechanisms of H_2_O_2_ and other electrophilic reagents are an important area of research that provides insight into how these reactive molecules are involved in the regulation of key signaling pathways and transcription factors. This has led to the establishment of the field of redox signaling, which overlaps with signal transduction and redox biology.

Oxidative stress (OS) has become a general term to describe the imbalance between the systemic expression of free radicals and the ability of cells to detoxify free radicals and counteract their damaging effects on proteins, lipids, and DNA [7,8]. While OS changes occur during aging, they are also essential for physiological homeostasis, and deviations from homeostasis in sustained redox signaling are now known to cause disease. An important relationship has been established between OS and various diseases, and this stressor is now at the forefront of research to elucidate pathogenesis. Indeed, OS has been linked to various diseases, including rheumatoid arthritis (RA) [9,10], Alzheimer’s disease (AD) [11,12], Parkinson’s disease (PD) [12,13,14], amyotrophic lateral sclerosis (ALS) [15,16], cardiovascular disease [17], allergies [18,19], immune disorders, metabolic dysfunction [20], diabetes [21,22], liver diseases [23], and cancer [24,25]. This paper presents a historical overview of redox biology and the nature of ROS and free radicals. We believe that this field can contribute to the prevention and treatment of future pathologies.

## 2. Earth’s History and the Evolution of Living Organisms

**Figure 1:**
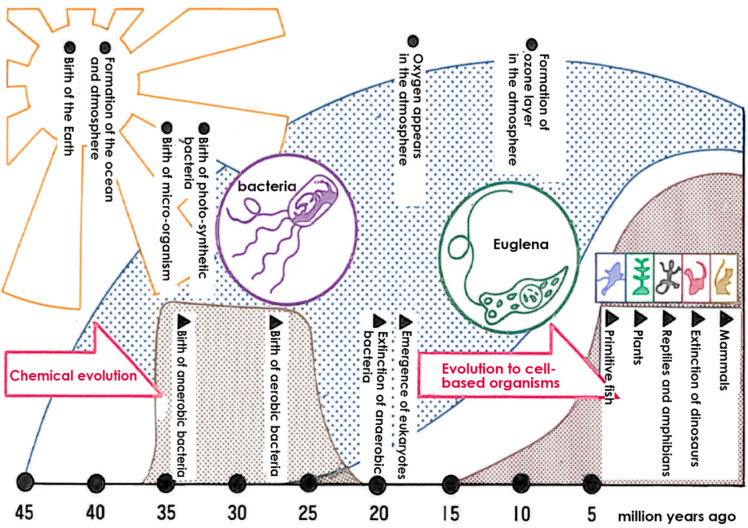
The Earth’s atmosphere and the evolution of living things. Anaerobic bacteria were the first to emerge, but the advent of oxygen caused the extinction of most anaerobic bacteria, and aerobic bacteria adapted to oxygen and cell-based organisms flourished.

The Earth’s history and the evolution of living organisms are intricately linked to the development of oxygen and its impact on biological systems. The Earth was formed approximately 4.6 billion years ago. The earliest microorganisms emerged about 3.9 billion years ago, followed by the birth of anaerobic bacteria. Subsequently, organisms capable of utilizing light for survival appeared, and approximately 2.5 billion years ago, oxygen, produced by photosynthetic bacteria, was released on Earth [26,27]. However, the emergence of oxygen led to the extinction of most anaerobic bacteria (Figure 1).

In contrast, aerobic bacteria evolved, and they survived by developing mechanisms to protect themselves from the harmful effects of oxygen-induced damage. Developing the ability to resist oxygen-related toxicity [28] resulted in the beginning of the history of aerobic organisms.

Following the generation of oxygen on Earth, the ozone layer was formed in the atmosphere, and this protected living organisms from powerful ultraviolet rays from space [29]. As a result, organisms were shielded from intense ultraviolet radiation, allowing for evolution on the Earth’s surface and the emergence of human beings.

It can be said, therefore, that the current diversity of life on Earth, as well as the survival of the human species, would not be possible without the ozone layer blocking the powerful ultraviolet rays from space. Therefore, the formation of the ozone layer has enabled the survival of humankind. Consequently, the potential consequences of the ozone layer being depleted by fluorocarbon gases [30], which is a global concern, poses a significant threat of extinction to various forms of life on Earth due to the damage caused by reactive oxygen species (ROS) and free radicals resulting from ultraviolet radiation. Protecting living organisms from this intense radiation in outer space is a crucial challenge in space exploration. As such, research on ROS and free radicals is indispensable for space travel.

**Figure 2:**
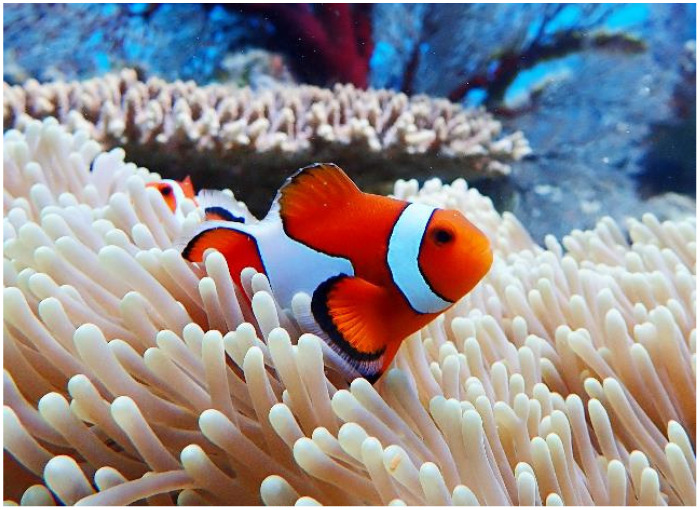
Highly pigmented tropical fish. The colorful natural pigments of tropical fish serve to scavenge free radicals and protect the fish from the ultraviolet irradiation (Photo by Shin-ichi Ohama, Diving shop Marine Mate, Ishigaki Island, Okinawa, Japan).

On the other hand, organisms in tropical seas are exposed to intense ultraviolet radiation but protect themselves from this hazard with powerful reactive active oxygen and free radical scavenging mechanisms. For example, corals and tropical fish, like Ocellaris clownfish (Figure 2), exhibit bright red and yellow colors. Ocellaris clownfish are well known for their body coloration, which is enhanced by the intake of the red carotenoid astaxanthin, a widely recognized antioxidant, in their diet. While the vivid body colors obtained with such pigments are beautiful, they are primarily responsible for the free radical scavenging effect. These organisms in effect use a variety of natural pigments to protect themselves from free radical damage [31].

## 3. Active Oxygen and Free Radicals

**Figure 3:**
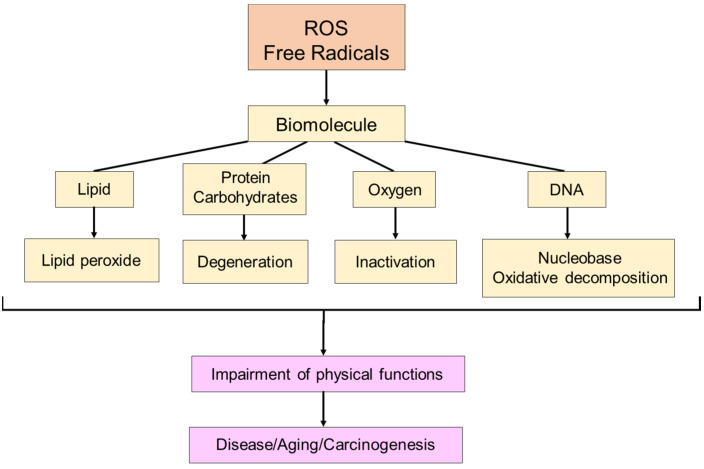
The effect of free radicals on living organisms. Free radicals damage biomolecules, such as lipids, proteins, carbohydrates, oxygen, and DNA, contributing to disease, aging, and cancer in living organisms.

**Figure 4:**
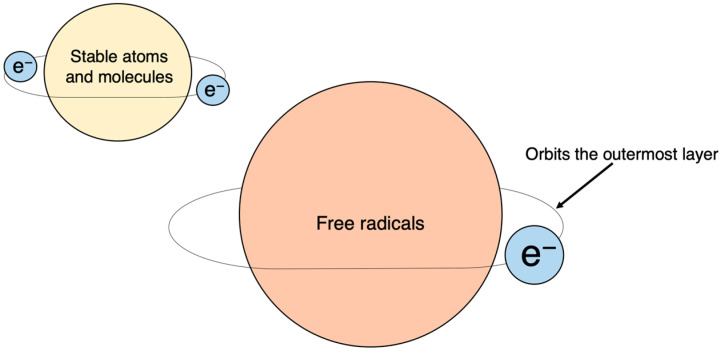
Free radicals: atoms or molecules with unpaired electrons. Electrons normally exist in pairs, with two in each orbital; unpaired electrons, with only one electron in each orbital, are chemically very unstable.

The discovery of SOD by McCord and Fridovich in 1969 marked a significant milestone in understanding the scavenging of superoxide (O**^·^**_2_^−^), a type of active oxygen and free radical [4]. This discovery paved the way for identifying various previously unknown chemical, biological, and biochemical reactions associated with O**^·^**_2_^−^, and other ROS and free radicals, as well as their biological damage mechanism and their implications for different diseases [32,33] (Figure 3 and Figure 4).

## 4. Definition of ROS

**Figure 5:**
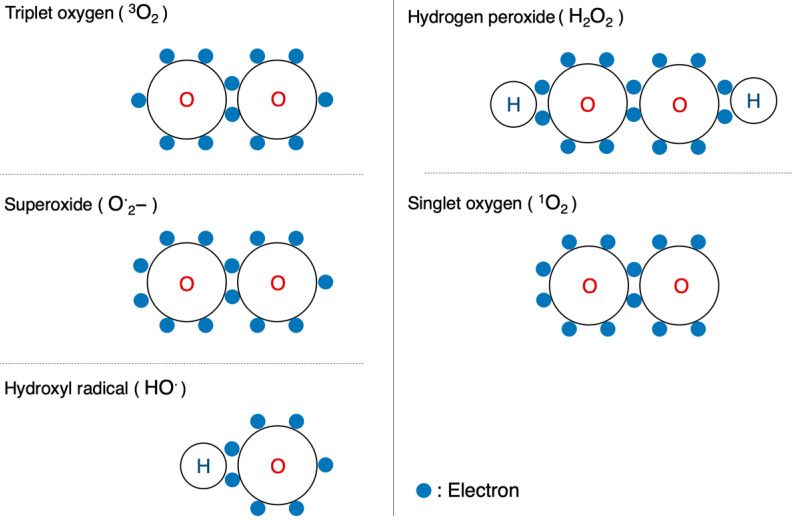
Configuration of oxygen and ROS. Schematic diagrams of the electron configurations of typical molecules are shown.

Oxygen is the most well-known free radical. Atmospheric oxygen possesses two unpaired electrons and is relatively stable on its own. Living organisms, however, convert this oxygen into more reactive forms, known as ROS. These are utilized in substrate oxidation reactions and oxygen addition reactions. Reactive oxygen, a derivative of atmospheric oxygen, encompasses several ROS, including O**^·^**_2_^−^, H_2_O_2_, hydroxyl radical (HO**^·^**), and singlet oxygen(^1^O_2_). Among these four, O**^·^**_2_^−^ and HO**^·^** are free radicals, while the other two are not (Figure 5). These species, on their own, or by cross-reacting with other species, can induce toxicity by attacking different cellular components, such as proteins, lipids, and nucleic acids [34,35], leading to various toxic effects.

## 5. Definition of Free Radicals

Atoms and molecules have a stable configuration when their outermost shell contains a paired set of electrons. However, when an electron is unpaired, the atom or molecule becomes a free radical. Unpaired electrons are unstable and highly reactive, as they constantly seek to form pairs, which makes free radicals highly reactive. Chemical bonds consist of two electrons that can be broken in two ways. The first involves one electron from each pair separating and moving to one side (A-B→A^+^ + :B−), resulting in each carrying a respective charge called a cation or anion. The second way occurs when the two electrons in a bond split symmetrically, leading to the creation of a free radical (AB→A^·^ +^·^B).

## 6. Generation of Active Oxygen and Free Radicals

ROS and free radicals are generated in our immediate environment. For instance, air pollution in the form of NO^·^ and NO_2_ is constantly present. Tobacco smoke, another pollutant, contains numerous free radicals [36,37]. Additionally, commonly used herbicides, including the well-knowns paraquat, produce free radicals to eliminate weeds and occasionally cause poisoning when ingested by humans [38].

Furthermore, ultraviolet and ionizing radiation also generate free radicals. Both types are utilized in cancer radiation therapy as well as in sterilization lamps. Anti-cancer drugs, such as adriamycin, bleomycin, mitomycin C, cisplatin, and neocarzinostatin generate free radicals to kill cancer; however, the free radicals they produce also cause side effects [39,40,41,42,43,44,45,46,47,48].

Radiation is also used to sterilize imported food, and, while it may not be common knowledge, drying laundry outdoors is another example of benefiting from the effects of free radicals. Hypochlorous acid, which is often used as a sterilizing bleach to disinfect medical operating rooms and toilets, transforms into free radicals and possesses a bactericidal effect. Viewed from this perspective, humans cannot avoid the effects of free radicals.

**Figure 6:**
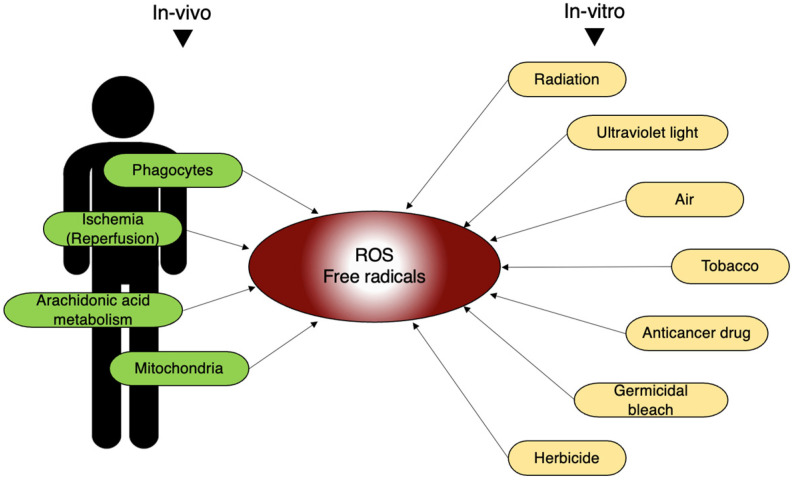
In vivo and in vitro sources of free radicals. In living organisms, ROS and free radicals are generated by various internal and external stimuli mentioned here.

In addition to the external environment, free radicals are also generated in living organisms. Free radicals are mainly produced by phagocytes, so called “eating cells”, centered on neutrophils. Phagocytes engulf foreign substances, such as bacteria, and generate ROS to eliminate them from the body [49,50]. Moreover, physiological processes, such as ischemia-reperfusion and arachidonic acid metabolism, also lead to the production of a large amount of ROS [51,52,53]. They are produced during the metabolism of arachidonic acid. Physiologically, free radicals are constantly generated within the mitochondria; however, in healthy mitochondria, they tend not to leak out (Figure 6).

## 7. Physiological Role of ROS and Free Radicals

Nitric oxide (NO^·^), a free radical, plays a crucial role in dilating blood vessels and maintaining blood flow as an endothelial-derived vaso-relaxing factor (EDRF). However, the radical nature of NO^·^ leads to its rapid interaction with other radicals, especially O**^·^**_2_^−^, resulting in the production of highly toxic peroxy-nitrite (ONOO^−^).

NO^·^ + O**^·^**_2_^−^ → ONOO^−^

**Figure 7:**
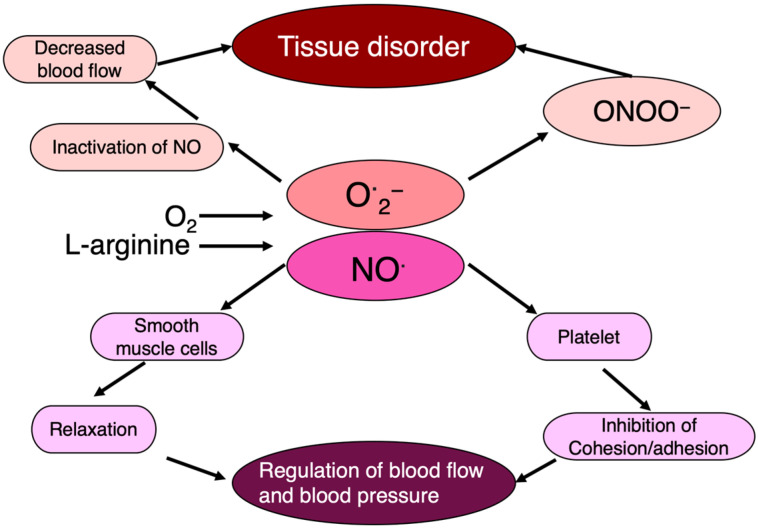
NO^·^ regulates blood flow, blood pressure, and tissue damage. NO^·^ regulates endothelial cell function as an endothelium-derived relaxing factor, in which O^·^2− is intimately involved.

In this way, when the NO^·^ effect is lost due to O**^·^**_2_^−^, highly toxic ONOO^−^ is simultaneously produced. This indicates that in addition to its own effects, O**^·^**_2_^−^ also regulates the action of NO^·^. This interplay between NO^·^ and O**^·^**_2_^−^ underscores the regulatory effects of free radicals on physiological processes, and the generation of free radicals and their physiological implications highlight the intricate balance between their beneficial and detrimental effects in various biological systems (Figure 7).

SOD regulates the tension state of vascular smooth muscle by relaxing blood vessels. In addition, NO^·^ is involved in the dilation and movement of the stomach and intestines. Hence, NO^·^ and its regulatory factor, active oxygen, such as O**^·^**_2_^−^, may control a variety of physiological reactions.

## 8. Utilization of Active Oxygen and Free Radicals

**Figure 8:**
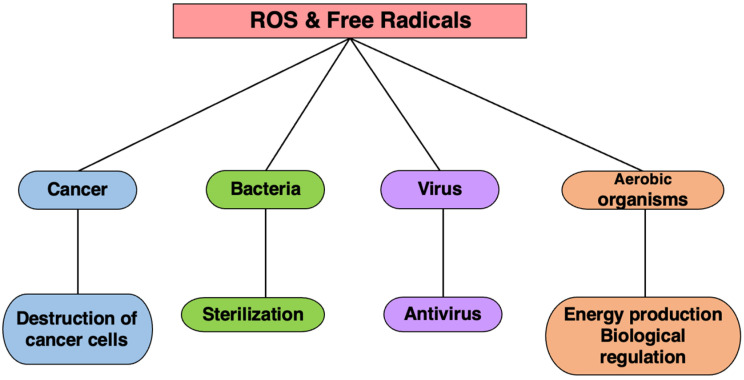
The effects of ROS and free radicals. Living organisms utilize the toxicity of ROS and free radicals to protect themselves from cancer, microorganisms, viruses, etc.

Living organisms harness the toxicity of ROS and free radicals to combat various threats. Phagocytic cells, such as neutrophils, employ these species as potent weapons to eliminate not only bacteria but also cancer, a significant threat to the body. During bacterial infections, activated neutrophils release large amounts of ROS to enhance bacterial eradication. Furthermore, the toxicity of these species is leveraged in cancer hyperthermia therapy, where elevated temperatures of 42–43 °C shrink tumors [54,55]. However, reducing neutrophils and scavenging ROS diminishes this anti-tumor effect. Thus, organisms utilize the toxicity of free radicals as a mechanism to eliminate foreign substances from the body (Figure 8).

## 9. Neutralization of Active Oxygen and Free Radicals

Aerobic organisms, such as humans, possess antioxidant functions in various internal and external compartments of their tissues and cells, to safeguard themselves from oxidative damage induced by free radicals and ROS [4]. This defense mechanism comprises three tiers: (1) preventive antioxidants that suppress the formation of free radicals [56], (2) radical-scavenging antioxidants that rapidly eliminate generated free radicals [54], and (3) reparative/generative mechanism for repairing and regenerating DNA, lipids, and proteins damaged by free radicals. Table 1 shows primary antioxidant mechanisms.

While these antioxidant mechanisms are effective, they are not infallible. When excessive free radicals are generated due to OS or weakened antioxidant mechanisms, the body suffers oxidative damage from free radicals, leading to various pathological conditions. While there are other antioxidant systems, the primary ones are the O**^·^**_2_^−^ scavenging, the H_2_O_2_, and the lipid peroxide scavenging systems.

## 10. Understanding Oxidative Stress

What is the real meaning of OS in terms of how it negatively affects living organisms? Stress, originally a term rooted in physics, refers to the “internal distortion that occurs within an object when subjected to external stimuli”. This concept was adapted to biological reactions by Canadian endocrinologist Hans Selye in the 1950s, who defined stress as an internal state in an organism that responds to external factors, leading to a disturbance of the organism’s homeostasis, and used the term “stressors” for the external factors that instigate stress. Due to the discovery of ROS and other factors, the term “oxidative stress” is currently used to describe the phenomenon that creates oxidative environments in living organisms due to external factors. In the context of living organisms, OS arises from the imbalance between free radicals and antioxidants, due to various external factors including physical, scientific, or a complex combination of both as depicted in Table 2.

Reduction indicates a reaction in which a molecule receives electrons, while oxidation involves a reaction where a molecule releases electrons, and this often occurs when oxygen (O _2_) binds to a molecule.

While animals require oxygen to produce energy, ROS, a byproduct of this energy metabolism, are produced during the conversion of oxygen. Under normal conditions, this reactive oxygen is efficiently removed by antioxidant enzymes and antioxidants. However, when the antioxidant mechanism malfunctions, ROS accumulates in the body, becoming a stressor and leading to a state of OS. In other words, OS may be considered as a state in which the mechanism for maintaining oxidative and reductive states within the body has failed.

This state is widely believed to contribute to the progression of various diseases, including Alzheimer’s disease [11,12], Parkinson’s disease [12,13,14], diabetic complications [21,22], and neurodegeneration caused by motor neuron disease [15]. However, in many cases, it is not clear whether oxidants instigated the disease or whether they are generated secondarily from tissue damage stemming from the onset of the disease. While the exact role of oxidants in disease onset is not always clear, OS has garnered significant attention as a factor in human diseases and has been the focus of extensive research.

Antioxidants are being studied and utilized as both medicinal and nutritional supplements for disease prevention and health maintenance. For example, several research has focused on the treatment of stroke and ischemia-reperfusion injury [57,58]. On the other hand, many compounds have been commercialized as nutritional supplements, and antioxidants are widely used to support health or to prevent malignant tumors and heart disease.

## 11. Evolution of Antioxidants

The term “antioxidant” was first coined in the late 19th century, initially referring to chemical species that inhibited oxygen consumption. Subsequently, antioxidants found widespread application in various chemical industries to prevent metal corrosion, control rubber crosslinking reactions, and inhibit fuel deterioration due to oxidative polymerization in internal combustion engines [59].

The specific biochemical roles of antioxidants became clearer in the mid-20th century as understanding of in vivo biochemistry and molecular biochemistry advanced. Many biological substances, whose importance became apparent through subsequent research progress, were rediscovered as antioxidants. For instance, the discovery of α-tocopherol (vitamin E) as a critical factor for healthy pregnancy, as evidenced by infertility in mice fed a diet deficient in α-tocopherol, highlighted its role as an antioxidant [60,61].

Such biochemical discoveries have significantly influenced nutrition and food chemistry, leading to the development and use of a plethora of naturally derived antioxidants as nutritional supplements to prevent food deterioration and enhance mineral absorption. Notably, some antioxidants, such as vitamin C and E, are consumed as food additives rather than as treatments for vitamin deficiencies [60,61].

In the medical field, the relationship between ROS, OS, and various diseases has garnered substantial attention. OS has been implicated in aging [33,62] and various conditions, such as neurological damage after recovery from cerebral ischemic disease [63] and the exacerbation of atheroma deposits via an inflammatory response by lipid peroxides in arteriosclerosis [64]. Consequently, antioxidants are being explored for their potential in treating stroke, arteriosclerosis, and slowing down the aging process. One such example is the development of Twendee X, a nutritional supplement with potent levels of eight key antioxidants that is being considered for application in several ailments, such as ischemic stroke and early dementia [65,66,67,68,69,70].

## 12. The Role of Antioxidants in Living Organisms

The role of antioxidants in living organisms is multifaceted. Antioxidants serve to eliminate reactive oxygen, free radicals, and their by-products from the body, thereby preventing harmful reactions derived from oxygen.

Additionally, a series of enzymes known as antioxidant enzymes catalytically decompose and metabolize antioxidants, rather than directly reacting with active oxygen and free radicals. These enzymes exhibit substrate specificity, meaning that different enzymes are involved depending on the type of ROS molecule. Moreover, multiple enzymes use a single ROS molecule as a substrate, and their presence in the body varies depending on the type of enzyme. For instance, H_2_O_2_, a type of ROS, is decomposed into water and oxygen by the enzyme catalase, while another enzyme, glutathione peroxidase, utilizes glutathione as a substrate to metabolize H_2_O_2_ to water. Furthermore, SOD decomposes O**^·^**_2_^−^ into water and H_2_O_2_.

Antioxidants can be classified based on their functional roles into the following categories:

(1) Antioxidants that prevent or suppress the production of ROS and free radicals.
(2) Antioxidants that scavenge and stabilize ROS and free radicals.
(3) Antioxidants that detoxify, eliminate, and repair and regenerate any damage caused by harmful substances in the body.

Table 3 shows the defense systems of living organisms against oxidative damage based on their functional roles. The main sites of active oxygen generation are considered to be the mitochondria in animals [71] and chloroplasts in plants [72,73]. Both are electron transport systems with metals as the center of enzymatic activity, and oxidase complexes effectively repeat redox, forming the basis of energy metabolism. However, where oxygen is used in the metabolic energy synthesis mechanism in these electron transport chains, O**^·^**_2_^−^ is generated as a secondary reaction. O**^·^**_2_^−^ generated in this way is immediately decomposed into water and H_2_O_2_ by manganese-SOD, a SOD present in mitochondria. Notably, Cu/Zn-SOD in the cytoplasm has a similar function.

Living organisms have adapted to the OS caused by ROS through chemical evolution, leading to the production of various antioxidant substances. For example, as part of the evolution from marine organisms to terrestrial animals, terrestrial animals began to produce antioxidants that are not found in marine organisms, such as ascorbic acid (vitamin C), alpha-tocopherol (vitamin E), polyphenols, and flavonoids.

Antioxidant compounds, such as vitamin C and vitamin E, are known to independently and selectively inhibit harmful reactions involving oxygen. These antioxidants are often found in low-molecular-weight form and function to stop ROS and free radicals. When such low-molecular-weight antioxidants directly react with ROS and free radicals, the selectivity of the reaction is low, and various oxidants react with the antioxidants.

Additionally, various oxidases exist in living organisms, some of which metabolize active oxygen itself as a substrate, while others decompose and metabolize harmful peroxides generated in living organisms. Furthermore, there are enzymes that convert compounds, such as vitamin C and vitamin E, that have reacted with oxidants into oxidizing agents and return them to their reduced forms. Many of these oxidases are considered antioxidants; they function using electron acceptors, such as glutathione and vitamin C, as substrates. That is, the presence of an antioxidant as a reducing agent is essential enzymes to metabolize peroxides. Many of these oxidases have iron, copper, manganese, and selenium atoms in their active centers. These metal ions are easily susceptible to redox reactions. It has also shown that circadian rhythm, a physiological oscillation referred to as body’s clock, also plays an important role on the OS defense by regulating glutathione levels [74].

## 13. Mechanism of Oxidative Stress Injury Caused by Active Oxygen and Free Radicals

The dynamic character of ROS and free radicals in living organisms is intricate, and the internal mechanisms (redox mechanisms) that control the generation and elimination systems in a well-balanced manner are often not fully understood. The relationship between diseases and OS reactions is significant [9,10,15,17,18,20,75] (Table 4), with approximately 100 types of diseases caused by active oxygen and free radicals; this number is expected to increase in the future. Many of these diseases are major contributors to mortality and have a substantial impact on human lifespan.

## 14. Active Oxygen/Free Radicals and Living Organisms Bio-Regulation

Enzyme systems that generate ROS and free radicals are broadly categorized into various metabolic systems: energy, nucleic acid, immune response, amino acid and protein, and lipid and carbohydrate (sugar) and drug metabolisms. In aerobic organisms, these enzymes are essential for maintaining “homeostasis”, the function that ensures that the state of the organism, for example temperature, is maintained regardless of changes in the internal or external environment.

The metabolic mechanism that maintains homeostasis itself generates ROS and free radicals, contributing to the decline in biological functions associated with aging. Thus, in the context of OS research, understanding the mechanisms that regulate the production of ROS and free radicals, as well as the antioxidant mechanisms that control these quantities, is crucial.

Antioxidant enzyme systems in living organisms play a vital role in suppressing the excessive production of ROS and free radicals. Therefore, it is vitally important to understand the role of both antioxidant enzymes within the body and those derived externally.

The continuous production of free radicals in aerobic organisms must be balanced by antioxidant mechanisms to mitigate the impact of aging [76] and maintain homeostasis [77]. Many aerobic organisms use oxygen to grow, reproduce, and exist throughout their entire life cycle. It is also inevitable that biological functions decline due to ROS and reactive nitrogen species generated from our reliance on oxygen [62]. Aging is defined as a decline in biological functions due to advancing age [62,78], and the decline can also be associated with a decrease in antioxidant capacity.

Many enzymatic reactions in living organisms work to maintain homeostasis. For example, they work to process waste produced in cells due to purine metabolism, amino acid metabolism, lipid metabolism, sugar metabolism, nucleic acid metabolism, and urea metabolism processes. As such, the lifespan of waste in aerobic organisms is determined by oxygen metabolism, which is genetically encoded in the organism.

Human homeostasis is composed of cells and tissues designed with nucleic acids and generated by the factors and proteins necessary for human growth. Essential amino acids, essential minerals, and essential fatty acids for example, are essential for our survival. Many antioxidants are produced by plants as a defense against OS. For humans, vitamins are particularly important.

Preserving youth and increasing life expectancy is an eternal challenge for humans. Many aerobic organisms use oxygen to grow, reproduce, and live their entire lives. Regulating the interplay of ROS and free radicals [79] may have an impact on the human lifespan [11].

## 15. Physical and Scientific Factors That Generate Active Oxygen and Free Radicals

Humans maintain homeostasis under various natural environmental conditions. Therefore, OS research cannot be discussed without considering natural conditions. Temperature, atmospheric pressure, and other physical factors are thought to be involved in the activation of oxygen, nitrogen, water, and other substances. Although X-rays, radiation, and ultraviolet radiation are electromagnetic waves that are harmful to humans, they can, directly or indirectly, kill pathogens, such as microorganisms and viruses, and play an important role in preserving the living environment. In this way, ROS and free radicals assist in maintaining homeostasis by killing or sterilizing microorganisms.

**Figure 9:**
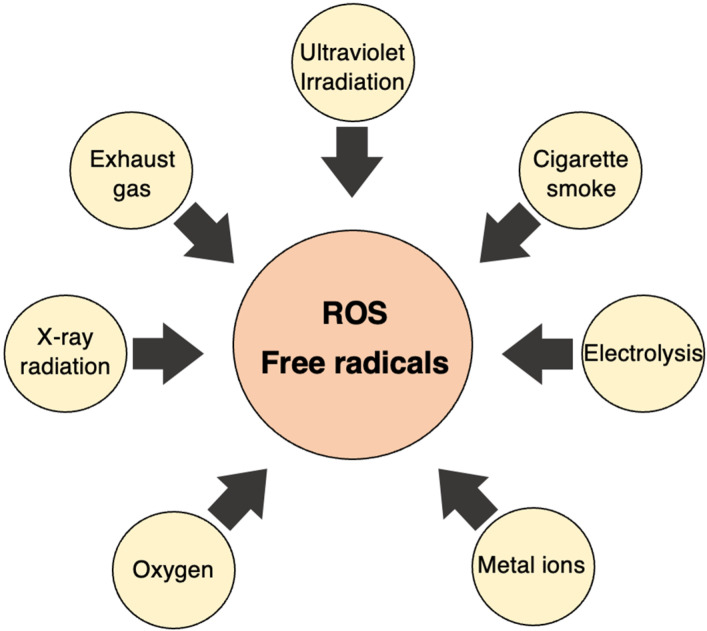
The physical and scientific factors involved in activating oxygen. Oxygen can become ROS/free radicals due to various factors, such as chemicals and radiation.

Naturally, from a physical and scientific perspective, there are several environmental factors that impact human health. As shown in Figure 9, active oxygen and free radicals are generated by physical factors, such as ultraviolet rays, infrared rays, ultrasound, and high temperatures mediated by gas molecules, such as oxygen and nitrogen. Conversely, the systems that generate active oxygen and free radicals have wide applications in such areas as environmental and biological fields. Understanding the interplay between these factors and human health is essential for developing strategies to mitigate their potential negative impacts and leverage their beneficial aspects for human wellbeing.

## 16. Role of Reactive Nitrogen Species in Triggering Oxidative Stress Responses

Various compounds in which nitrogen and oxygen are combined are usually called nitrogen oxides, and reactive nitrogen species (RNS) are a family of antimicrobial molecules derived from NO^·^. RNS is a radical containing one unpaired electron. Nitrogen dioxide (NO_2_) is called an active nitrogen species and is also a free radical. In living organisms, nitric acid tends to be the most reactive among the nitrogen species.

NO^·^ is a colorless and odorless gas and an atomic molecule at room temperature, but at low temperatures the proportion of dimeric N_2_O_2_ increases and it converts to N_2_O_2_ to liquid and solid forms.

Although NO^·^ contains one unpaired electron, it is a relatively stable radical and has low reactivity. Even when NO^·^ is produced in living organisms, it has lower reactivity and a longer lifespan than other radicals. NO^·^ does not react with other chemically stable compounds in living organisms. However, when it is paired with a radical or a metal ion, it triggers a physiological response and NO^·^ exhibits strong reactivity.

### 16.1. Production of Nitric Oxide In Vivo

The human body typically produces NO^·^ from L-arginine using the enzyme nitric oxide synthase (NOS). There are two types of NOS: constitutive NOS (cNOS), which is always present in cells and produces NO^·^ when necessary, and inducible NOS (iNOS), which is not normally present but can be activated by stimuli, such as endotoxin and inflammatory cytokines. cNOS is further classified into two types according to the locale of its function, namely endothelial NOS (eNOS) and neuronal NOS (nNOS), which were originally identified in vascular endothelial cells and neuronal cells, respectively. These three NOS isoforms (eNOS, nNOS, and iNOS) have been found in numerous other cells since they were first discovered (Table 5).

The reaction between NO^·^ and O**^·^**_2_^−^ in the body is caused by an enzyme (Cu/Zn-SOD that decomposes O**^·^**_2_^−^. This has been shown to extend the lifespan of NO^·^ when added to the tissues in which it is produced.

NO^·^ + O**^·^**_2_^−^ → ONOO^−^

This reaction is quite rapid, and peroxynitrite (ONOO^−^) is usually produced when NO^·^ and O**^·^**_2_^−^ interact.

### 16.2. Effects of Nitric Oxide on Living Organisms

NO^·^, a simple diatomic gas, plays a multifaceted role within the body. It is involved in systemic regulation, particularly in the vascular system, where it is produced by various types of NOS. The main types of NOS involved in the vascular system are a constitutive type, present in vascular endothelial cells, and an inducible type, present in smooth muscle cells. The most important physiological action of NO^·^ in the vascular system is to dilate blood vessels and regulate blood pressure.

Macrophages, immune cells responsible for phagocytosis, contain iNOS. High levels of NO^·^ generated by iNOS are used to combat foreign entities, such as pathogens or tumor cells. However, excessive NO^·^ production is associated with diseases, such as hypertension, dyslipidemia, atherosclerosis, heart failure, coronary artery spasm, and impotence. Thus, the amount of NO^·^ produced in the body must be carefully regulated, as both excessive and deficient levels are linked to various diseases.

Septic shock, a condition characterized by systemic infection due to the invasion of pathogens via the bloodstream, triggers the production of excessive NO^·^ by macrophages and macrophage-like cells. In this condition, iNOS produces NO^·^ in macrophages and macrophage-like cells throughout the body to attack pathogens. This state is a normal defense reaction; however, an overproduction of NO^·^ relaxes vascular smooth muscles causing blood vessels to dilate and a sudden drop in blood pressure that leads to a state of shock (septic shock). Therefore, the precise regulation of NO^·^ levels is crucial for maintaining homeostasis and preventing disease.

## 17. Conclusions

ROS and free radicals play important roles in homeostasis in living organisms. They injure living cells and tissues and also promote enzyme production and cell signaling. NO· is a free radical, the action of which is indispensable for plants and animals on earth. Many recent studies have shown that NO· performs diverse biological functions, while it has been subject to NOX regulation as a poisonous gas. Understanding the nature of ROS and free radicals and their physiological mechanism of regulation will lead to the prevention of diseases and extension of our lifespan.

## Figures and Tables

**Table 1:** In vivo mechanism of antioxidants.

Preventive mechanism	Hydroperoxide reduction of H_2_O_2_	Glutathione peroxidase, catalase, glutathione -S- transferase, peroxidase
	Metal ions chelation	Transferrin, ferritin, ceruloplasmin, lactoferrin, albumin
	Singlet oxygen quenching	Beta-carotene, bilirubin
Radical scavenging mechanism	Captures radicals in lipid layer	Vitamin E
	Captures radicals in the water layer	Vitamin C, uric acid, bilirubin
	Captures O^·^_2_^−^	SOD
Repair and regeneration mechanism	Phospholipase A2 Protease AMP-activated protein kinase Endonuclease	

**Table 2:** Categories and causes of physiological and psychological stress.

Physiological stress	Physical stress	Temperature, humidity, light, sound, ultraviolet rays, radiation, atmospheric pressure
	Chemical stress	Oxygen, pH, osmotic pressure, metal ions, ethanol, environmental pollutants (nitrogen oxides, sulfur oxides, etc.), hazardous chemicals
	Combined stress	Starvation, aging, inflammation, infection, allergy, trauma, ischemia
	Daily life stress	Family illness and death, divorce, moving, debt, childbirth, entrance exams
Psychological stress	Occupational stress	Human relations, office transfer, reassignment, promotion
	Other stress	War, natural disaster

**Table 3:** Defense against oxidative damage in living organisms.

(1) Preventive antioxidants Inhibits the production of active oxygen and free radicals	(a) Non-radical decomposition of H_2_O_2_ and lipid peroxide catalase	Glutathione peroxidase Glutathione S-transferase
	(b) Chelation and inactivation of metal ions	Transferrin Lactoferrin Ceruloplasmin Albumin
	(c) Elimination and inactivation of active oxygen	SOD Carotenoids
(2) Radical scavenges Scavenges active oxygen and free radicals to suppress or stop chain reactions.	(a) Water-soluble	Vitamin C Uric acid Bilirubin Albumin
	(b) Lipid-soluble	Vitamin E Ubiquinol Carotenoids Vitamin A
(3) Repair and regeneration function		Lipase, Protease, DNA repair enzyme, Acyltransferase

**Table 4:** A range of diseases attributed to reactive oxygen and free radicals.

Type of Disease	Disease
Malignant tumor	Stomach cancer, lung cancer, colon cancer, gallbladder cancer, liver cancer, bladder cancer, prostate cancer
Aging	Stains, wrinkles, decreased bone density, hearing loss, gray hair, aging odor
Cardiovascular disease	Ischemic heart disease, aortic aneurysm, arrhythmia, arteriosclerosis obliterans
Endocrine/metabolic diseases	Diabetes, Graves’ disease
Kidney disease	Acute nephritis, chronic nephritis, nephrosclerosis
Hepatobiliary pancreatic disease	Hepatitis, cholecystitis, pancreatitis
Neurological disease	Ischemic brain disease, epilepsy, Alzheimer’s dementia, encephalitis, meningitis, Parkinson’s disease
Collagen disease	Rheumatism, systemic lupus erythematosus, systemic scleroderma, mixed connective tissue disease
Respiratory disease	Respiratory diseases pneumonia, asthma, interstitial pneumonia, acute lung injury

**Table 5:** The role of NO^·^ radical and how it is generated.

Relaxation of vascular smooth muscle Inhibition of platelet aggregation and adhesion	Vascular endothelial cells Platelet
Involved in intercellular communication by increasing cyclic GMP	Brain cells, nerve cells Renal epithelial cells Adrenal gland, tumor cells
Relaxes smooth and skeletal muscles through neural stimulation	Esophagus, stomach, small intestine Cerebral artery, mesenteric artery Bronchus Corpora cavernosa/index muscle Anococcygeus muscle
Target cell damage	Macrophage Polymorphonuclear leukocytes Liver Kupffer cells

## Data Availability

Not applicable.
